# Assessment of IgG antibodies to *Pseudomonas aeruginosa* in patients with cystic fibrosis by an enzyme-linked immunosorbent assay (ELISA)

**DOI:** 10.1186/s13000-014-0158-z

**Published:** 2014-08-22

**Authors:** Renan Marrichi Mauch, Cláudio Lúcio Rossi, José Dirceu Ribeiro, Antônio Fernando Ribeiro, Marcos Tadeu Nolasco da Silva, Carlos Emílio Levy

**Affiliations:** Department of Clinical Pathology, Faculty of Medical Sciences, State University of Campinas, Rua Alexander Fleming 105, Block FCM 12, 2nd floor, Postal code: 13083-881 Campinas, SP Brazil; Department of Pediatrics, Faculty of Medical Sciences, State University of Campinas, Campinas, SP Brazil

**Keywords:** *Pseudomonas aeruginosa*, Cystic Fibrosis, Serology, ELISA

## Abstract

**Background:**

The usefulness of serological tests for detection of *P. aeruginosa* pulmonary infection in cystic fibrosis (CF) is controversial. Here, we assessed the value of detecting anti-*P. aeruginosa* IgG by a quantitative enzyme-linked immunosorbent assay (ELISA) for identification of *P. aeruginosa* infection in patients with cystic fibrosis.

**Methods:**

Serum concentrations of anti-*P. aeruginosa* IgG were assessed in 117 CF patients classified according to their *P. aeruginosa* colonization/infection status (never colonized; free of infection; intermittently colonized and chronically infected) and in 53 healthy subjects by the ELISA test standardized with the St-Ag:1–17 antigen.

**Results:**

The rate of IgG seropositivity and the median of IgG concentrations of this antibody in patients chronically infected were significantly higher than those found in the other CF groups and in the healthy control group.

**Conclusion:**

Detection of anti-*P. aeruginosa* IgG can be an useful tool for identification of *P. aeruginosa* chronic infection in patients with CF.

**Virtual Slides:**

The virtual slide(s) for this article can be found here: http://www.diagnosticpathology.diagnomx.eu/vs/13000_2014_158

## Background

Cystic fibrosis (CF) is the most common hereditary disease in the Caucasian population, with a wide range of clinical and genetic variants [1]. The genetic defect occurs in the CF transmembrane conductance regulator (CFTR) gene, which codes for a protein that regulates the transport of electrolytes across epithelial cell membranes. Mutations in the CFTR gene affect sodium and chloride ion transport, resulting in the disruption of the ionic composition and volume of airway surface fluid. This fluid is normally thin to allow removal of inhaled microorganisms via ciliary action; however, in the presence of CFTR mutations, it increases in volume and becomes viscous, clogging the airways. As a result, microorganisms entering the distal airways are not cleared and can cause chronic infections with progressive inflammation and respiratory insufficiency [2]. The clinical outcomes are similar to those found in diffuse panbronchiolitis, chronic obstructive pulmonary disease and idiopatic pulmonary fibrosis [3-5].

*Pseudomonas aeruginosa* is an important pathogen in nosocomial and opportunistic infections due to its high intrinsic resistance to antibiotics and ability to develop multidrug resistance, which lead to serious therapeutic problems [6]. *P. aeruginosa* is the most important and frequent pathogen in CF patients [2,7,8], responsible for elevated morbidity and mortality [1-3]. When chronic *P. aeruginosa* pulmonary infection is established, this bacterium is practically impossible to be eradicated; however, the elimination from the airways is possible by early intervention with antibiotic therapy, as soon as the pathogen settles in the organism. Thus, the prompt treatment is recommended and may delay the progression of the pulmonary disease [9].

Detection of *P. aeruginosa* in the diagnostic routine is made mostly through sputum culture; however, many patients - especially children under seven years old- are incapable of producing an expectorated sputum specimen. Bronchoalveolar lavage (BAL) is an option, but is invasive and usually employed only when there is a compelling reason to obtain a respiratory sample and other approaches have failed [10,11]. More superficial samples include oropharyngeal (OP) and cough swab [12,13]; however, the frequent insufficient samples obtained through swabs can lead to false negative results and recent reports have shown that OP swabs poorly reflect the lung microbiota [13,14]. The difficulty in obtaining representative respiratory specimens from the airways of infants and children indicates the need for the use of methods that can complement or be an alternative to microbiological culture [15].

Serological tests have been used to assist the identification of *P. aeruginosa* infection in CF patients, particularly in patients who do not produce sputum. Some reports also suggest that serological tests may help in the differentiation between intermittent colonization and chronic infection [16,17].

The diagnostic value of serological tests for detection of *P. aeruginosa* infection in cystic fibrosis is controversial, with large variations in the sensitivity and specificity values [18]. The aim of the present study was to assess the value of detecting anti-*P. aeruginosa* IgG by a quantitative enzyme-linked immunosorbent assay (ELISA) for identification of *P. aeruginosa* infection in patients with cystic fibrosis.

## Methods

### Patients, controls and serum samples

Serum samples were obtained from 117 patients with confirmed CF diagnosis [19-21] who attended the cystic fibrosis ambulatory of the University Hospital, State University of Campinas, São Paulo, Brazil. Of these 117 patients, 35 (22 male, 13 female; median age = 3.0 years) had never been colonized with *P. aeruginosa*, 27 (8 male, 19 female; median age = 8.2 years) were free of infection, 24 (16 male, 8 female; median age = 9.0 years) were intermittently colonized and 31 (17 male, 14 female; median age = 14.7 years) were chronically infected by the pathogen. Fifty-three serum samples from pediatric patients and university students (32 male, 21 female; median age = 9.7 years) without CF and no previous history of *P. aeruginosa* infection were used as controls.

### Bacteriology and classification of the patients according to microbiological culture

The lower airway secretion was obtained by sputum expectoration in an universal sterile container. In case of non-expectorating patients, secretion of the upper airways was collected with OP swab and transported to the laboratory within 3 h. Microbiological culture and identification were performed according to methodology previously described [7]. To classify the CF patients according to their *P. aeruginosa* colonization/infection status, we used an adaptation of the Leeds criteria [8], basing on the results of 12 last respiratory microbiological cultures prior to the blood sample collection, considering cultures were made every three month. Thus, we classified the patients as: (1) **chronic infection**, when ≥ 50% of the cultures were positive to *P. aeruginosa*, (2) **intermittent colonization**, when < 50% of the cultures were positive to *P. aeruginosa*, (3) **free of infection**, when the last 12 cultures were negative to *P. aeruginosa* with a previous positive culture and (4) **never colonized**, when *P. aeruginosa* was never isolated in microbiological culture.

### Determination of optimum assay conditions

The ELISA was standardized by using excess amounts of all reagents except the one being tested. A pooled human standard antiserum (Statens Serum Institute, Copenhagen, Denmark) diluted 1:200 was arbitrarily designed as having 1000 activity antibody units per milliliter (U/mL). Artificial serum standards containing different antibody concentrations (U/mL) were prepared by diluting this antiserum with phosphate buffered saline containing 0.1% Tween 20 (PBS-T). For the St-Ag:1–17 antigen (Statens Serum Institute, Copenhagen, Denmark) and conjugate (peroxidase-labeled rabbit anti-human IgG, Dako, Glostrup, Denmark) titrations, dilutions from 1:250 to 1:64000 were tested. The linearity of substrate conversion was assayed at time intervals from 1 to 60 min, using serum standards containing 25 and 100 U/mL.

### ELISA for detection antibodies to P. aeruginosa

The antigen preparation diluted 1:2000 in 0.1 M carbonate-bicarbonate buffer, pH 9.5, was used to sensitize wells of Maxisorp ELISA plates (Thermo Fisher, Waltham, MA, United States). After sensitization for 1 h 30 min at room temperature (RT), the wells were washed three times with PBS-T, after which 100 μl of 0.1% bovine serum albumin in 0.1% PBS-T were added to the wells. After incubation for 1 h at RT, the wells were washed twice with PBS-T and 100 μl of each serum sample diluted 1:800 in PBS-T were added in triplicate to the wells. After a further 1 h incubation at RT and washing three times with PBS-T, 100 μl of conjugate diluted 1:4000 in PBS-T were added to the wells and the plates were incubated for 1 h at RT. After incubation and five washes with PBS-T, 100 μl of substrate system (0.42 mM tetramethylbenzidine and 1.42 mM H_2_O_2_ in 0.1 M sodium acetate/acetic acid buffer, pH 5.5) were added to the wells. Ten minutes after the addition of the substrate, 100 μl of 2 N H_2_SO_4_ were added to each well to stop the color reaction and the resulting absorbances were read at 450 and 630 nm in an ELISA reader (Labsystems, Helsinki, Finland). A negative control and artificial positive serum standards containing different concentrations of the antibody were included in each ELISA plate. The final optical density for each well was determined by subtracting the mean optical density of two antigen controls in the corresponding plate. All serum samples and positive standards were tested in triplicate and the mean activity determined. A standard curve was used to translate optical density readings of each serum specimen into U/mL.

### Definition of the results status

The cut-off value for the assay was determined by a receiver operating characteristic (ROC) curve, using the ELISA results obtained with CF patients chronically infected with *P. aeruginosa* and healthy subjects. Analyzing the results obtained with different dilutions of serum samples from chronically infected patients, a variation of 5% was applied to the cut-off value to define three areas of test results - positive, negative and a grey zone between them.

### Statistical analyses

The Chi-square test was used to compare the rates of IgG seropositivity among the CF patients and healthy controls simultaneously; a confidence interval of 95% was adopted. With respect to the medians of the antibody concentrations, the Mann–Whitney test and the Kruskal Wallis test were used for comparisons between two groups and more than two groups, respectively. The Spearman test was used to identify correlation between antibody levels and age of CF patients chronically infected with *P. aeruginosa*. In all tests, a *p value* < 0.05 was considered statistically significant. The statistical analyses were done using SAS (Statistical Analysis System) for Windows version 9.2 (SAS Inc., Cary, NC, United States).

## Results

Titration experiments defined 1:2000 and 1:4000 as the optimal dilutions of antigen and conjugate, respectively. The linearity studies showed that the substrate conversion rates were linear for at least 10 min for standards containing 25 and 100 U/mL. The cut-off value for specific *P. aeruginosa*-IgG antibodies determined by the ROC curve was 14.57 U/mL. Applying a 5% of variation to this cut-off, negative, grey zone, and positive test results were defined as < 13.8 U/mL, 13.8 to 15.3 U/mL and > 15.3 U/mL.

**Figure 1:**
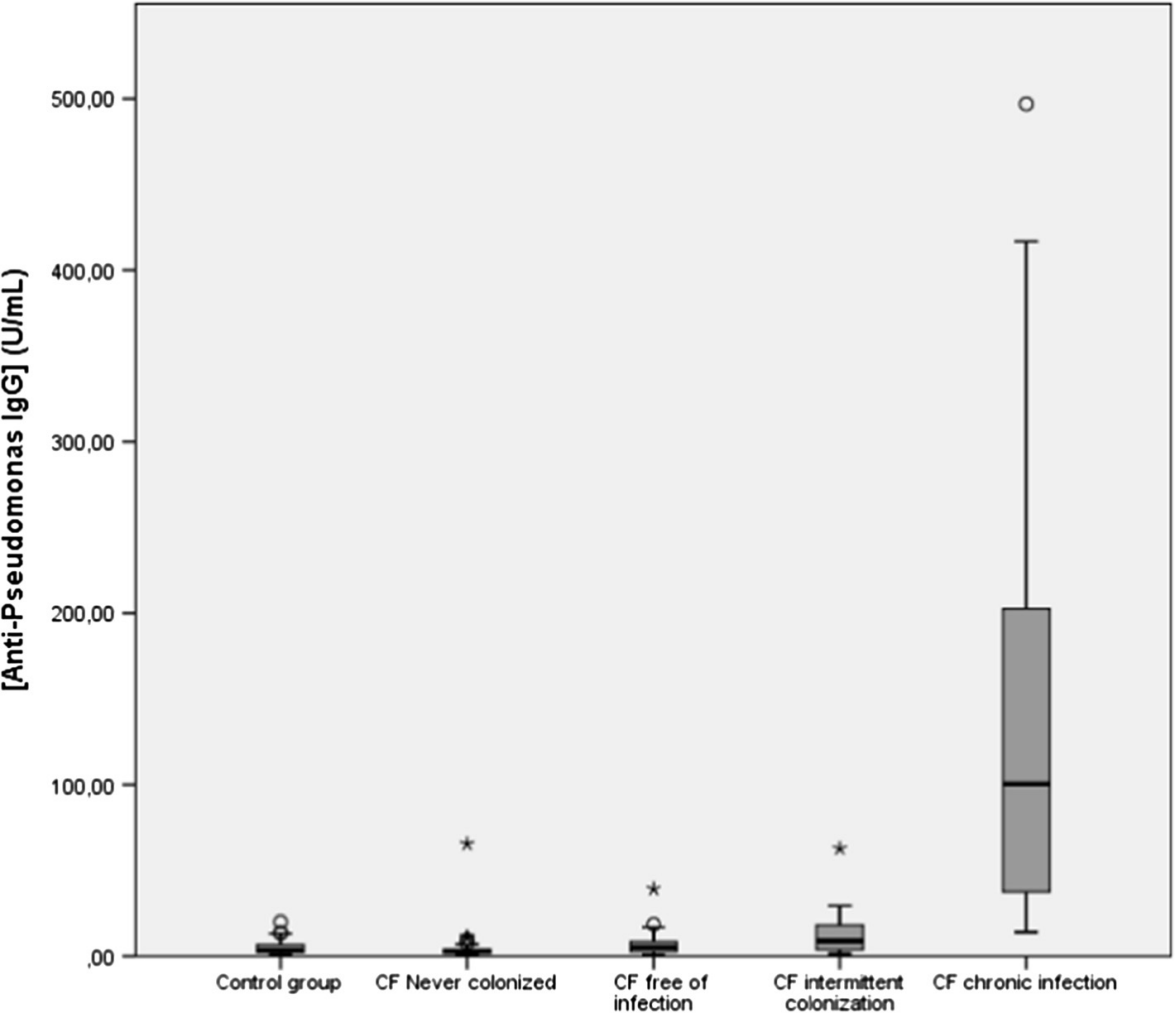
**Serum concentrations of anti-** ***P. aeruginosa*** **IgG (U/mL) in patients classified according to their** ***P. aeruginosa*** **colonization/infection status and in healthy controls.** The median of IgG concentration in patients chronically infected was significantly higher than those found in the other CF groups and in the healthy controls (*p < 0,0001*). No statistically significant differences were found between CF patients intermittently colonized and free of infection, between CF patients free of infection and the control group and between CF patients never colonized and the control group.

A total of 170 serum samples, 117 from CF patients and 53 from healthy subjects, were assayed for anti-*P. aeruginosa* IgG. Table 1 presents the demographic characteristics of CF patients and healthy controls, as well as the rate of positivity for anti-*P. aeruginosa* IgG in all groups. Positive results were found in 1/35 (2.9%) of the CF patients never colonized, in 3/27 (11.1%) of patients free of infection, in 8/24 (33.3%) of patients intermittently colonized, in 30/31 (96.8%) of patients chronically infected and in one healthy control (1.9%). The rate of IgG seropositivity in patients chronically infected was significantly higher than those found in the other CF groups and in the healthy control group (p < 0.0001). The rate of IgG seropositivity in intermittently colonized CF patients was significantly higher than those found in CF patients never colonized and in the healthy controls (p < 0.001). Figure 1 shows the medians of IgG concentrations (U/mL) in CF patients and healthy controls. The median of IgG concentration in patients chronically infected was significantly higher than those found in the other CF groups and in the healthy control group (p < 0.0001). No significant differences were found between healthy controls and CF patients free of infection (p = 0.232), between healthy controls and CF patients never colonized (p = 0.062) and between CF patients free of infection and those with intermittent colonization (p = 0.064). A positive correlation between antibody levels and age was found in chronically infected CF patients by the Spearman test (r = 0.40; p < 0.01).

**Table 1:** **Gender, age and detection of anti-** ***P. aeruginosa*** **IgG in patients with cystic fibrosis and in healthy controls**

**Parameters**	**Subject groups**				
	**CF chronic infection**	**CF intermittent colonization**	**CF free of infection**	**CF never colonized**	**Healthy controls**
**Number of subjects**	31	24	27	35	53
**Gender (M/F)**	17/14	16/8	8/19	22/13	32/21
**Median age - years (range)**	14.7 (3 – 30)	9.0 (1.6 – 16)	8.2 (3 – 25)	3.0 (0.16 – 26)	9.7 (0.16 – 30)
**% IgG seropositivity [C.I. 95%]**	96.8 (81.5 – 99.8)	33.3 (16.4 – 55.3)	11.1 (2.9 – 30.3)	2.9 (0.2 – 16.6)	1.89 (0.1 – 11.4)

The rate of IgG seropositivity in patients chronically infected was significantly higher than those found in the other CF groups and in the healthy control group (*p < 0.0001*). The rate of IgG seropositivity in intermittent colonized patients was significantly higher than those found in CF patients never colonized and in the healthy controls (*p < 0.001*).

## Discussion

The antibody response to *P. aeruginosa* in cystic fibrosis has been considered a marker of chronic infection, inflammation and tissue damage [22,23]. In the last three decades, several studies have shown the potential of antibody detection for early diagnosis of *P. aeruginosa* pulmonary infection [15-18] in order to initiate the early eradication therapy [23].

The standardization of a quantitative ELISA for detection of specific antibodies requires that the concentrations of all reagents and reaction times be optimized. It is important the measurement of enzymatic activity during the linear part of the reaction, when substrate concentration is much higher than enzyme concentration. The standardization of the assay involves the use of a positive standard from which a standard curve is prepared and included in each ELISA plate, with the reactivity of the samples being tested determined from the standard curve. The utilization of a standard curve in every reaction plate reduce the variability of the assay [24,25].

In our study, the rate of anti-*P.aeruginosa* IgG seropositivity and the concentration of this antibody were significantly higher in the patients chronically infected than those in the other CF groups, corroborating previous studies that showed high frequency of detection and elevated levels of this antibody in this phase of infection [18,26,27]. One serum sample from a CF patient with chronic infection by *P. aeruginosa* who was under antibiotic therapy gave a negative serological result for the pathogen. Some studies have shown that antibiotic treatment regimens may result in decrease of the antibody levels to *P. aeruginosa* [28-30].

A healthy control with no history of *P. aeruginosa* infection showed a positive serology to the pathogen. Previous reports showed the occurrence of false negative results [22,31-33]. Since *P. aeruginosa* is an environmental pathogen, it is possible that this healthy control have got in contact with this bacterium in a period next to the study, leading to a transient antibody response [23,26]. Some authors have also shown that the St-Ag:1–17 preparation contains antigens which are cross-reactive with other gram-negative bacteria, including *Haemophilus influenzae* [17,28], which is a common microorganism in the respiratory tract of healthy children and adults. Høiby *et al*. [22] and Pedersen *et al*. [33] observed, in healthy controls, an increase in the antibody levels over the age. They attribute these facts to the normal occurrence of cross reactions with other pathogens and to a possible acquired immunity over time, after patient contact with *P. aeruginosa*.

It has been known for many years that chronic *P. aeruginosa* infection is preceded by an intermittent colonization with the pathogen and previous reports showed an overlap in the antibody levels between chronically infected and intermittent colonized CF patients [16,26,28]. In the present study, we did not find statistical difference in the antibody titers between the intermittently colonized and the free of infection group. Indeed, the majority (66.7%) of intermittently colonized patients had negative serology to *P. aeruginosa*; however, it is well known that *P. aeruginosa* isolation in respiratory samples not necessarily indicates infection, unless there is a specific humoral response [8,34]. In some patients, the colonization can persist for 1 to 2 years without eliciting an humoral immune response [33], which can be due to the presence of transient or non pathogenic strains, to the inadequate immune response to the pathogen [28], or even to the adequate immune response of the patient through secretory IgA [23,28]. Besides, there is a great interchange of patients between these two groups when longitudinal microbiological data are observed [26]. On the other hand, the positive antibody levels of 33.3% of the intermittently colonized patients in the present report may suggest a change in the colonization/infection status for chronically infected [16,17]. These results draw attention to limitations in the Leeds criteria for definition of colonization and infection status [8], which are based solely on microbiological cultures. Serology results, in conjunction with more frequent respiratory cultures, can play an important role in elaborating a better definition for colonization and infection, mainly for patients classified as free of infection and intermittently colonized [16,17,26,35].

Among the CF patients with negative microbiological respiratory culture, the rate of seropositivity was 2.9% and 11.1% in the never colonized group and the free of infection group, respectively. Such findings may suggest limitations in respiratory culture, which may not reflect the lung microbiota [11,13,14], or even recurrent infection [27,34]. Many of these patients are not sputum producers, making more difficult to obtain representative respiratory samples, which may lead to false negative results. So, these groups constitute an important focus of interest for detection of serum anti-*Pseudomonas* antibodies [29].

A large variation in the performances of immunological assays used for the diagnosis of *P. aeruginosa* infection in CF patients has been observed [18], which is expected and is related to several factors including the antigen chosen, the criteria used for selection of the patients and healthy controls, the criteria adopted for classification of the colonization/infection status, the immune status of the patients at time of blood collection, the antibiotic treatment regimen, the intrinsic properties of the serological methods used for antibody detection, the antigen preparation, the lack of an appropriate reference standard and the method for calculating the cut-off value [18,30,36]. Here, we considered that culture results of one year prior to the serological analysis were not enough to provide a consistent classification of *P. aeruginosa* colonization/infection status. So, we used the 12 last culture results, seeking to optimize this classification. For classification according to serological results, we fixed negative and positive cut-off values through a ROC analysis, based on the results of a healthy population. This may help to avoid bias when determining the accuracy of the method [30,36]. The St-Ag:1–17 antigen was chosen by the fact of being a commercialized antigen; besides, the studies with the St-Ag:1–17 has been continuous over the last 30 years. Other antigens, like exotoxin A, elastase and alkaline protease are quorum-sensing regulated antigens [17] and require a higher bacterial load to elicit the immune response. Since St-Ag:1–17 constitutes a pool of 64 antigens from 17 different *P. aeruginosa* serotypes, the immune response is earlier in relation to these other antigens [17,23].

## Conclusions

This report has the limitations of a cross-sectional study. Since that only one serum sample was obtained from each patient, we could not take further conclusions. Longitudinal reports have demonstrated the capacity of serology to detect *P. aeruginosa* infection until three years before microbiological culture [18]. Future protocols based in longitudinal studies in our laboratory should verify the importance of our findings. Besides, other diagnostic resources must be evaluated, like real-time PCR of respiratory samples, culture of paranasal sinuses secretions, the humoral response to other antigens and the secretory IgA response. Overall, the results of the present study show that the ELISA for detection of anti-*P. aeruginosa* IgG can be an useful tool for identification of *P. aeruginosa* chronic infection in patients with cystic fibrosis.

### Ethical approval

The study was approved by the Ethics Committee (reference number: 719/2009) of the Faculty of Medical Sciences, State University of Campinas, in accordance with the resolutions of the Brazilian Ethics Committee.

## Abbreviations

BAL: Bronchoalveolar lavage
CF: Cystic fibrosis
OP swab: Oropharyngeal swab
ELISA: Enzyme-linked immunosorbent assay
PCR: Polymerase chain reaction
PBS-T: Phosphate buffered saline containing 0.1% tween 20
RT: Room temperature
ROC: Receiver operating characteristics
St-Ag:1–17: Standard pooled antigen (17 different *P. aeruginosa* serotypes)
U/mL: Activity antibody units per milliliter
